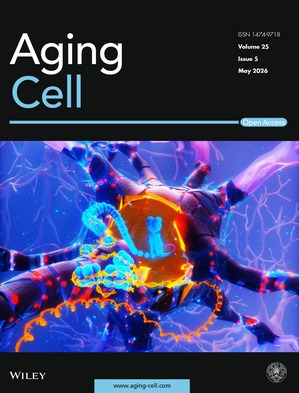# Additional Cover

**DOI:** 10.1111/acel.70549

**Published:** 2026-05-13

**Authors:** Cristian Gerónimo‐Olvera, Stephen M. Scheeler, Carlos Galicia Aguirre, Genesis Vega‐Hormazabal, Daniela Garcia, Long Wu, Natalia Murad, Kevin Schneider, Kenneth A. Wilson, Nikola T. Markov, Sicheng Song, Jesse Simons, Akos A. Gerencser, Emily Parlan, Sean D. Mooney, Eric Verdin, Judith Campisi, Tara E. Tracy, David Furman, Simon Melov, Lisa M. Ellerby

## Abstract

Cover legend: The cover image is based on the article *Exceptional Longevity Modifying Allele *APOE2* Promotes DNA Signaling Pathways Resisting Cellular Senescence in Human Neurons* by Cristian Gerónimo‐Olvera et al., https://doi.org/10.1111/acel.70494.